# The effects of community participation program on smoke-free homes in a suburban community of Thailand

**DOI:** 10.18332/tid/133876

**Published:** 2021-05-10

**Authors:** Peeraya Suteerangkul, Sunee Lagampan, Surintorn Kalampakorn, Naruemon Auemaneekul

**Affiliations:** 1 School of Nursing, Eastern Asia University, Pathumthani, Thailand; 2 Department of Public Health Nursing, Faculty of Public Health, Mahidol University, Bangkok, Thailand

**Keywords:** community participation, smoke-free home, negotiation skills, smoking ban, emotional support

## Abstract

**INTRODUCTION:**

Smoking inside the home affects the health of both the smoker and family members via secondhand exposure. This research examined the impact of a community participation program on creating smoke-free homes in a suburban community in Thanyaburi district, Pathumthani province in Thailand.

**METHODS:**

The study involved families, with a smoker in the home, that were randomly assigned to intervention and control groups each containing 27 families. The intervention group was administered with the community participation program for smoke-free homes for 5 sessions during the 6-month period of study. The program included providing information on secondhand smoking and harms, knowledge about quitting smoking and healthcare support, practice skills, campaigns in the community, visiting and encouraging, and reflecting and evaluation. The control group was normally treated by the community committee and health volunteers. Data collection was undertaken at baseline and at 6 months after implementation by an interview with questionnaires.

**RESULTS:**

Our results show that after the implementation, the intervention group reported significantly higher mean score on skills in negotiating with smokers for a smoking-ban inside home and mean score on emotional support for non-smoking inside the home than those at baseline and those of the control group. The proportion having smoking ban home rules in the intervention group was significantly higher than at baseline and that of the control group (92.6% vs 18.5%). The proportion of smoke-free homes was higher in the intervention than in the control group (75% vs 0%).

**CONCLUSIONS:**

These findings suggest that community participation programs for smoke-free home may be effective in raising awareness on the impact of secondhand smoke among family members and in working together to manage smoke-free home environments. The program may be applicable for further development within communities to achieve smoke-free homes.

## INTRODUCTION

Secondhand smoke (SHS) is a major public health problem^1^ and commonly noted at home^2,3^. Exposure to secondhand smoke is significantly associated with the prevalence of hypertension, angina, and stroke^4^. In the US, economic costs attributable to smoking and exposure to secondhand smoke now approach $300 billion annually^5^. Regulating smoking inside households is challenging since the home is a private place^6^. Recent research has also reported that no smoking inside the home was associated with education level, particularly among higher educated adults^7^. Populations may be unaware of the dangers of secondhand smoke^8^. Smoking has become a misleading value that passes on from smoking parents to the youth in the home^9^.

In Thailand, the Royal Thai government had enacted the Tobacco Control Act, combined with the Health Care Act for non-smokers, but the target was not reached as cigarette consumption was not affected by a tax increase^10^. A previous study showed the prevalence of smokers among Thai adults was 45.6% for men and 3.1% for women^11^. Even if additional tobacco control measures have successfully reduced smoking in public areas, SHS exposure at home is still a public health problem as the legislation could not cover personal areas. Additionally, it was not clear if a law prohibiting secondhand smoke exposure at home would come into effect in 2020^12^.

To improve the indoor air environment, smoking bans in the entire household should be implemented^13-15^. The primary issue is that smokers may be unable to quit smoking^16^. The solution often focuses on particular groups, and previous research results usually emphasized only increasing awareness^16^. Within family interventions, family members may have failed to participate and have lacked the negotiation skills to support smokers’ behavioral change, especially regarding a smoker who may be the household head. Negotiation skills are essential to solving problems in human life^17^. Effective tobacco control programs should therefore include a focus at the level of the community, helping community leaders and family members to promote their skills in negotiating^6^ with smokers, campaigns for smoke-free home environments, and community participation, so as to strive for sustainable community development. Exposure to secondhand smoke inside the home is an urgent issue to be resolved and requires the collaboration of all sectors including local agencies, community committees and family members, as well as smokers who need to cooperate with the community.

Aligning with the objectives of Healthy People 2020^18^ to reduce the problem of secondhand smoke and increase the number of smoke-free homes, this research aimed at examining the effects of a community participation program on implementing smoke free-homes within a suburban community in the Thanyaburi district of the Pathumthani province in Thailand.

## METHODS

### Design and setting

This study had a quasi-experimental research design using a two-group pretest-posttest design. The population included families of smokers who resided in the respective area of the Thanyaburi district of the Pathumthani province^19^.

### Participants

Participants were recruited by: 1) purposive sampling from 1 community in each of the 2 subdistricts with similar characteristics under the local administration of Thanyaburi municipality (two communities had strong community leaders and supported program implementation until end of the program); 2) simple random sampling, with one community as a control group and another as intervention group (lottery sampling); and 3) randomly selected families with a smoker in the home to obtain intervention families and control families. The sample size was calculated by G*Power analysis based on the effect size of 0.80, Z_α_=1.96 (value of error α of 0.05), β=0.20, and power of the test (1-β)=0.80, which led to a sample size of 26 in each group. To take into account potential sample loss, the sample size was increased by 15% to 30 persons in each group.

We advertised to each community for families that met the inclusion criteria and voluntarily could participate in the research. The inclusion criteria were: families residing for at least 6 months in the community and having family members living with a smoker in the same home (father, mother, child or relative), aged ≥18 years, with no history of narcotics use, or having severe stage chronic disease or neuropsychological disorders. Exclusion criteria were: participants who refused to give information and did not wish to join the study, who moved to another community, or had an accident or serious health problem.

Clarification was made to the participants about their participation and they were asked to sign a consent form to voluntarily participate in the program. Participants were free to withdraw from the program at any time. At the end of the program at 6 months, 27 families remained in the intervention group because two families moved out of the area to other provinces, and in one family the smoker had died. In the control group, 27 families also remained as three families moved out of the area to other provinces.

Two research instruments were employed, a program developed by the community committee based on participation in the Community Participation Program for a Smoke-Free Home (Supplementary file Table S1), and a data collection instrument. Other materials and media used for the program were a Smoke-free Home Kit, which contained smoke-free home pamphlets and tobacco-related illness brochures, Pico Smokerlyzer®, digital blood pressure equipment, and a handbook. Storyboards were created by a community volunteer and researcher on the topic of ‘Tobacco Danger’ and showed to the community.

### Data collection instruments

Data collection involved a questionnaire on general characteristics of the family including sex, age, education level, family status, congenital disease, family relationships, and presence of smoking-ban home rule/regulation. Structured interview form was constructed by the researcher and consisted of the following parts:

Part 1: Negotiation with the smoker, measured by 10 items on four-rating scales. These involved asking family members about their behaviors related to listening to the current smokers and behavior towards the current smokers, explaining the objective, giving reasons and choices for setting up smoking ban rules together, and keeping good relationships within the family. The total score was 40 with each item scored as follows: regular practice=4, often practice=3, sometimes practice=2, rarely practice=1, and the negative statements were reversed. The reliability of rating scales was analyzed and Cronbach’s alpha coefficient=0.89. Content validity index of negotiation for a smoking ban at home was 0.79.

Part 2: Emotional support for no smoking inside home, measured by 6 items on four-rating scales. These involved asking family members about their caring support, listening to smokers, helping, doing activities together, encouraging and appreciating smoking cessation inside the home, being concerned and giving love. The total score was 24, each item was scored as follows: regular practice=4, often practice=3, sometimes practice=2, rarely practice=1 and the negative statements were reversed. The reliability of rating scales was analyzed and Cronbach’s alpha coefficient=0.93. Content validity index of negotiation for a smoking ban at home was 0.73.

Smoke-free homes were assessed by two questions: 1) ‘Is there a total ban on smoking inside the home?’, and 2) ‘Is there anyone smoking inside the home or outside at fence?’. Responses were ‘yes’ or ‘no’.

### Data collection

The research was conducted from December 2016 to June 2017. The intervention group that followed the Community Participation Program for Smoke-Free Homes involved the five sessions (Supplementary file Table S1). The control group was normally handled by the community committee and health promotion volunteers and included the provision of advice on sources of service for smoking cessation and a campaign for a smoke-free home. At the end of the program (at 6 months), the research team educated the control group on smoke-free homes and the hazards of secondhand smoke and gave out a handbook on smoke-free homes and the dangers of tobacco in developing various chronic diseases. This research was approved by the Ethical Review Committee for Human Research, Faculty of Public Health, Mahidol University (COA. No. MUPH 2016-114). Data were collected by research participants conducted by the researcher and research assistants at two-time points at baseline, prior to project implementation at week one and after implementation at week 24.

### Statistical analysis

Descriptive statistics was used to calculate frequency, percentage, mean, and standard deviation. Chi-squared test was used to compare the distribution of background between groups. Paired t-test was used to determine the difference of mean score within group before and after project implementation. Test of the difference in mean scores between intervention and control groups was performed with independence t-test. Test of the difference in proportion of the presence of smoking-ban home rule and proportion of smoke-free home and dichotomous variables used Fisher’s exact test. A significance level for test of hypothesis was determined at 0.05.

## RESULTS

### General characteristics

Both sample groups were similar in characteristics with an age between 18 and 86 years (intervention group: mean age=49.9±18.8; control group: mean age=51.9±19.7). Most of the family members were female (81.5% in the intervention group; 85.2% in the control group); attained primary education (51.9% in the intervention group; 55.6% in the control group). Family members ranged 2–7 persons. The age of current smokers ranged from 18 to 82 years in the intervention group (mean=44.2 ±17.1) and 18–86 years (mean=42.4±20.6) in the control group. Most of the current smokers in the intervention and control group attended elementary and primary school (63% and 51.9%) and almost all were employees (77.8% and 63.0%). No significant difference between the two groups at baseline were identified (p>0.05) (Table 1).

**Table 1:** Comparison of characteristic of family members and current smokers in the intervention group and the control group

*Characteristics*	*Intervention group (n=27)*		*Control group (n=27)*		*χ^2^*	*p*
	*n*	*%*	*n*	*%*		
**Age of family members** (years)						
19–39	8	29.6	7	25.9	0.948	0.000
40–59	9	33.4	9	33.4		
≥60	10	37.0	11	40.7		
**Gender**					-0.199	0.320
Male	5	18.5	4	14.8		
Female	22	81.5	23	85.2		
**Relationship in family**					3.536	0.128
Harmony/unity	25	92.6	17	63.0		
Frequently/sometimes argue	2	7.4	10	37.0		
**Age of smoker** (years)					-0.073	0.717
19–39	10	37.0	12	44.4		
40–59	12	44.5	9	33.4		
≥60	5	18.5	6	22.2		
**Smoker’s education level**					-0.307	0.119
Less than elementary and primary school	17	63.0	14	51.9		
High school, college and higher	10	37.0	13	48.1		
**Smoker’s occupation**					-0.141	0.484
Governed/business/ agriculturalist	2	7.4	5	18.5		
Employee	21	77.8	17	63.0		
Housewifery/student	4	14.8	5	18.5		

### Negotiation for smoking ban inside the home

Before program implementation, the intervention and control groups had no difference in the mean score of negotiation for a smoking ban inside the home (p>0.05). After program implementation, the mean score of negotiation for a smoking ban inside the homes of the intervention group was significantly higher than that at baseline and that of the control group (p<0.05) (Table 2). The intervention and control groups had no difference in the mean score of emotional support for no-smoking inside home before program implementation (p>0.05). After program implementation, the mean score of emotional support for no-smoking inside the home of the intervention group was significantly higher than that at baseline and that of the control group (p<0.05).

**Table 2:** Comparison of family members negotiating for smoking ban inside home and emotional support for no-smoking inside home, between the intervention and control groups at baseline and after implementation

	*Intervention group*	*Control group*		
	*mean±SD*	*mean±SD*	*t-test*	*p*
**Family members negotiating for smoking ban inside home**				
At baseline	21.2±5.3	24.8±6.3	1.456	0.27
After	30.8±4.4	22.8±6.0	10.261	<0.001
**Emotional support for non-smoking inside home**				
At baseline	10.9±4.3	2.7±5.0	-1.57	0.129
After	17.8±4.1	11.4±4.6	5.62	<0.001

*p<0.05 significant.

### Smoking-ban rules at home

Before program implementation, there was no difference in establishing smoking-ban home rules between the intervention group and control groups (p>0.05). After program implementation, establishing smoking-ban home rules in the intervention group was significantly higher than that at baseline (92.6%) and that of the control group (18.5%) (p<0.05) (Table 3).

**Table 3:** Control of smoking-ban rules between the intervention and control groups at the baseline and after implementation

	*Intervention group (n=27)*		*Control group (n=27)*		*χ^2^*	*p*
	*n*	*%*	*n*	*%*		
**Smoking-ban rules** (at baseline)					0.13	1.000
Yes	4	14.8	5	18.5		
No	23	85.2	22	81.5		
**Smoking-ban rules** (after)					30.00	<0.001
Yes	25	92.6	5	18.5		
No	2	7.4	22	81.5		

*p<0.05 significant.

### Smoke-free home

After six months, in the intervention group, 21 current smokers began to smoke outside the home (75%). While in the control group, none smoked outside the home (0%). The number of smoke-free homes in the intervention group was higher than at baseline and that in the control group (Table 4).

**Table 4:** Control of smoke-free home between the intervention and control groups at the baseline and after implementation

*Smoke-free home*	*Intervention group (n=27)*		*Control group (n=27)*	
	*n*	*%*	*n*	*%*
**Smoke-free home** (at baseline)				
Yes	0	0	0	0
No	27	100.0	27	100.0
**Smoke-free home** (after)				
Yes	21	75.0	0	0
No	6	25.0	27	100.0

## DISCUSSION

The current results show that the community participation program constructed by a community committee and based on the concept of participation was effective in changing home environments, with the implementation of home smoking-ban rules by negotiation with the smokers at home.

Smokers’ family members were able to express opinions by using soft words at the right time. Talking with smokers should be sincere and caring for their health while convincing them to see the dangers of SHS to family members living in the same home. Family members were allowed to share opinion and establish smoke-free home rules and also encouraged to avoid any possible conflicts. Smokers’ families in suburban Thailand predominantly had a low level of education, and the majority were hired workers or housewives, which is consistent with previous studies reporting that smokers and their families have a low education level^7,20,21^. Previous research found that a factor associated with establishing smoke-free home rules was being a college graduate^22^. However, there was a good relationship in smokers’ families and smokers without conflict. This contradicted earlier research indicating that allowing for smoking inside the home created conflict^22^.

Similarly, when parents share the idea about reducing secondhand smoke in home environments, the number of cigarettes smoked in the home decreases^23^. Successful negotiation depends heavily on the personalities of both those making the request and those they are making it to^17,24^. Our study is in line with previous research reporting that children with skills to negotiate with adults to convince them not to smoke inside the home will be able to achieve a smoke-free home^25^.

Moreover, posting no-smoking signs inside the home is important to remind family members, encourage smokers, and support a smoke-free environment at home. Previous research has noted that a ‘No Smoking’ sign may help prevent exposure to secondhand smoke at home and help enforce smoking-ban home rules^8,26,27^. Using a ‘Smoke-Free Home’ sign functions as a visible positive force and notice for preventing smoking inside and has been found to be an effective strategy to increase voluntary smoke-free homes^2,26,28^.

With respect to emotional support for non-smoking inside the home, the community participation program for a smoke-free home can enhance emotional support for smokers to avoid smoking at home. The most effective social support is one unnoticed by the receiver and indicating a high level of emotional support to reduce smoking^23,29^. In contrast, there is an association between mood and smoking behavior, as smokers may increase their consumption of cigarettes when they feel happy or sad^30^. Thus, an attempt to quit smoking may fail due to the lack of assistance from their family^31^. Emotional support was associated with quitting smoking^29,32^.

The current study suggests that after the implementation of a community participation program for a smoke-free home, there was an increase in smoking-ban home rules which were established by family members and smokers, and about one-third of homes with smokers became smoke-free. This is in line with a previous study suggesting that only one-third of the participants reported having made their home smoke-free^2,33^. Therefore, establishing smoking-ban home rules requires collective agreement between family members and smokers to allow for participation and to create a smoke-free home environment. This is also similar to reports that smokers and non-smokers viewed that smoking should not be allowed in homes^2,34^. Moreover, previous research has indicated that households within communities may be willing to create smoke-free home environments and support ‘smoke-free home’ management (restricting smoking in spaces indoors or on verandas) which may increase the number of smoke-free homes in the community^21,30,35^.

This research was a community participation program for smoke-free homes in a suburban community, Thailand. The success of the program was due to the good cooperation of the community committee, and various strategies implemented for a smoke-free home environment. The current study recommends that public health nurses should implement the ‘Community Participation Program for Smoke-Free Homes’ and work with the community committee as a partnership for increasing smoke-free homes in their communities. The family is the key stakeholder who will support and make a rule in the house, so community committees should include smoke-free homes in the community agenda and support family members to achieve the goal.

### Limitations

This study has several limitations. Our community may not be representative of other urban communities. There may have been a spillover between communities, and there may have been self-report bias.

## CONCLUSIONS

This study showed that the community participation program for a smoke-free home effectively drives cooperation among family members and smokers to establish smoking-ban home rules and encourage smokers to smoke outdoors to manage a smoke-free home environment. This will guide personnel in developing smoke-free homes through the participation of the community and every family member.
