# Supplementary material for: The effects of community participation program on smoke-free homes in a suburban community of Thailand
Source: Tob Induc Dis. 2021 May 10;19:35. doi: 10.18332/tid/133876 (PMC8106388; doi:10.18332/tid/133876)
Supplement: Supplementary file 1 [file TID-19-35-s1.pdf]

Table S1. Community Participation Program for Smoke-Free Home comprising 5 sessions

Session	Week/Timing	Activities in the Program
1	Wk. 1/120min.	Activities involve dissemination of information about smoking situation in community; conclusion of opinions of community committee (composed of a head of community, a head of village healthcare volunteer, a head of district, 6 persons of village healthcare volunteer, and 6 persons of community committee), smokers' families and smokers regarding the problem issues of second-hand smoke in the home, attitudes, and guidelines for solutions; participation in making action plans, objectives, targets, and strategies to take actions; participation in formulating a policy of "smoke-free home in community".
2	Wk. 2/120min.	Community committee cooperated with the research team of professional registered nurses from community Hospital (Thanyaburi) in activities to educate about second-hand smokes in the home, skills in negotiating with smokers for smoking-ban inside home, establishing smoking-ban home rules, emotional support for non-smoking inside home, posting smoke-free home stickers to remind smokers of no smoking inside home, and advice on the sources of help for smokers wishing to quit smoking.
3	Wk. 3/3 hours/day-2 days	Community committee engaged in smoke-free home campaign in community, walk through survey in community, inspection of home environment, posting smoke-free home stickers/signs, installment of smoke-free home campaign board at the community entrance, and search of volunteers to quit smoking and refer to concerned agencies.
4	Wk. 1- 6/30-45 minutes per person	Community committee and research team visited families on the list, with community committee/community volunteers being responsible to take care of 4 – 5 families/person; provided encouragement and moral support; asked about problems and obstacles and solved the problems together for at least once per family.
5	Wk. 7/90 min.	Meeting for conclusion and evaluation; community committee provided recommendations, pointed out problems and obstacles in programme implementation, and worked together for action plans in community.

© 2021 Suteerangkul P. et al.
